# Recovery, completion and further referral after Improving Access to Psychological Therapies in Norfolk and Waveney

**DOI:** 10.1192/bjo.2025.10045

**Published:** 2025-07-07

**Authors:** Amanda Burke, Max Bachmann, Charlotte E. L. Jones, Julii Brainard, Zillur Rahman Shabuz, Alice M. Dalton, Rachel Cullum, Nick Steel

**Affiliations:** Norwich Medical School, University of East Anglia, Norwich, UK

**Keywords:** Remission, talking therapies, outcomes, young people, gender minority

## Abstract

**Background:**

Improving Access to Psychological Therapies (IAPT), an NHS England service providing talking therapies, is meeting its target recovery rate of 50%. However, engagement in treatment, as well as recovery rates, may be lower for some groups.

**Aims:**

To assess variation in treatment completion and recovery rates by demographic and socioeconomic group and to describe rates of further referrals for patients to IAPT and secondary mental health services.

**Method:**

Using 121 548 administrative records for 2019–2020 and 2022–2023 for the Norfolk and Waveney area, we estimated associations of age, gender, ethnicity and deprivation with the likelihood of treatment completion and recovery using logistic regression modelling. We also described rates of further referrals.

**Results:**

Younger people and those living in deprived areas were less likely to recover or complete treatment, with those aged 16–17 years (*n* = 735) having the lowest adjusted odds for recovery (adjusted odds ratio = 0.5, 95% CI: 0.5–0.6) compared with those aged 36–70 years, and those aged 18–24 years (*n* = 23 563) having the lowest rate of completion (adjusted odds ratio = 0.5, 95% CI: 0.5–0.6). Further referrals before April 2022 were recorded for 45.4% of 6513 patients who had completed treatment and 68.8% of 9469 who had not completed treatment, and for 39.4% of 2007 recovered patients in 2019–2020 and 53.1% of 1586 who had not recovered. Non-completers had relatively more further referrals to secondary mental health services compared with completers (43.6% *v*. 22.8%; *P* < 0.01).

**Conclusions:**

Younger people and those living in deprived areas have lower recovery and completion rates. Those who have completed treatment and not recovered have higher rates of further referrals.

Improving Access to Psychological Therapies (IAPT), more recently known as ‘NHS Talking Therapies’, is a National Health Service (NHS) programme established in England in 2008 to improve access to evidenced-based psychological therapies for individuals with depression and anxiety disorders. The 2019 NHS Long Term Plan set a target for 1.9 million adults per annum to have access to IAPT by 2023–2024. In 2022–2023, the number of referrals had already increased to 1.8 million.^1^ On 1 April 2024, access targets moved away from the number of referrals to the number of additional people completing a course of treatment.^2^

IAPT delivers psychological therapies recommended by the National Institute for Health and Care Excellence. In 2024, IAPT’s recovery rate target was 50%, with 53% to be reached by 2028–2029.^2^ Recovery is defined by the NHS as a change in scores on two validated mental health diagnostic questionnaires from above to below clinical thresholds.^2^ Recovery status is recorded from the second treatment session, meaning that the recovery rate statistic does not encompass all patients referred to IAPT or starting treatment, rather the subset of patients who have attended two or more sessions. In 2022–2023, there were 1.8 million referrals to IAPT; 1.2 million of the referred patients (69%) entered treatment and 0.7 million (38%) completed at least two sessions, of whom just under half recovered. Therefore, approximately 28% of all those who started treatment were recorded as having recovered, and approximately 19% of all those referred recovered.^1^

## Existing evidence around recovery and engagement with treatment

Lower engagement with IAPT treatment, such as non-attendance at appointments, has been associated with patient characteristics including higher baseline anxiety scores, younger age and increased deprivation.^3,4^ Higher engagement has been associated with patient self-referral, although rates of self-referral may be mediated by deprivation.^3–5^ As with engagement, recovery is associated with specific patient characteristics. Stochl et al (2022) found that patients with lower baseline symptom severity, less functional impairment and older age had a greater likelihood of achieving recovery.^6^ Verbist et al (2023) similarly found that lower recovery rates were associated with high baseline scores for anxiety and depression, as well as with long-term sickness or unemployment, being female or having a higher number of previous IAPT referrals.^7^ Previous research suggests that people from some minority ethnic groups experience lower recovery rates, but where deprivation was controlled for, the effect of ethnicity on recovery among IAPT patients was attenuated.^2,8^

## Evidence gaps around further referral rates and for youth aged 16–17 years

Numerous studies of IAPT services for adults have found immediate improvements in depression and anxiety measures post-treatment. Long-term outcomes, including further referral rates, remain less well understood.^9^ Further referral rates are of interest because mental health problems such as depression often recur. At least 50% of adults may have a second episode of depression after the first.^10^ Martin et al (2022) found that few studies had addressed whether IAPT prevents transition to secondary and acute care services and recommended that research should focus on dropout rates and further referrals among IAPT patients.^11^ A further evidence gap involves outcomes for young people, because most studies have focused on adults aged 18 years and over.

## Study aims

This exploratory study, which was commissioned as a service evaluation, addresses these gaps in the research literature by:

- investigating characteristics associated with recovery and treatment completion in a cohort that included both adults and young people aged 16–17 years, a group for whom evidence in the literature is especially limited; and
- describing further referral rates, i.e. any further referrals within a 4-year period to either IAPT or secondary mental health services for those recovered and not recovered, and for those who completed treatment and those who did not, in the absence of evidence about long-term outcomes for IAPT patients.

We hypothesised that patients who were younger or from more deprived areas might have lower recovery and treatment completion rates, on the basis of previous evidence about engagement with IAPT,^5,6^ and that those who did not complete treatment or recover would be more likely to be referred again.

## Method

We used administrative records to (a) investigate factors associated with recovery status and treatment completion status in IAPT, for example, associations between recovery status and age group; and (b) for individuals referred to IAPT in 2019–2020, analyse further referrals up to 31 March 2023 to both IAPT and secondary mental health services, also by recovery status and treatment completion status.

### Study population

The study population consisted of individuals aged 16 years and over, residing in the Norfolk and Waveney (N&W) area, for whom a referral was received by IAPT between 1 April 2019 and 31 March 2023. N&W is a health authority in eastern England, with a population total of around 1 million, consisting of the county of Norfolk and the Waveney district of Suffolk.

In the N&W area, both IAPT and secondary mental health services, which are separate, are provided by Norfolk and Suffolk NHS Foundation Trust. IAPT services are available to all people aged 18 years and over in England, and, in some areas, including N&W, also to young people aged 16 and 17 years. Secondary mental health services in N&W, which serve individuals with moderate to severe mental health problems, offer a children’s and young people’s service for persons aged up to 25 years, as well as adult services for older patients.

In Norfolk, 7.4% of lower layer super output areas (LSOAs; small geographic areas used for governmental statistics) are in the 10% most deprived nationally (most deprived decile); however, there are areas of intense deprivation, such as Great Yarmouth, where 24.6% of LSOAs are in the most deprived decile.^12^ In the Waveney district, 13.7% of LSOAs are in the most deprived decile.^12^ On the basis of 2022 mid-year population estimates, the proportion of young people in Norfolk is slightly lower than the national figure for ages 16–17 (2.0% *v*. 2.3% nationally) and ages 18–24 (7.6% *v*. 8.3% nationally); the corresponding figures for Waveney are 2.1 and 5.6%, respectively.^13^

### Study data

Health planning in the area is overseen by the NHS N&W Integrated Care Board (ICB). In this study, we used pseudonymised N&W ICB activity data-sets for referrals received by IAPT and secondary mental health services. Both data-sets included a record for each referral received for each patient during the study time period, comprising 121 534 records for referrals of 87 716 patients into IAPT and 104 621 records for referrals of 71 393 patients into secondary mental health services. Self-referrals made up 68.4% of all IAPT referrals, general practitioners’ surgeries comprised 16.2%, and the remaining 15.4% were from a mix of other sources including schools. Routes into secondary mental health services involved referral from an organisation rather than self-referral; this occurred predominantly through general practitioners (66.7%), with other referrals coming from sources such as hospital accident and emergency services (11.3%) and secondary healthcare (5.9%). Variables included gender, ethnicity and the LSOA of the patient’s home address. The LSOA was used to identify the Index of Multiple Deprivation 2019 decile of the patient’s home address, because lower levels of engagement in treatment and recovery have been associated with increased deprivation.^4,12,14^ The databases also included referral date, referral reference number and patient reference number.

The outcome variables used in the statistical analysis of IAPT data were recovery status (recovered or not) and treatment completion status (completed treatment or not). The completed treatment variable was dichotomised from N&W IAPT database variable ‘referral outcome’, which is the NHS Data Dictionary variable ‘Discharge from improving access to psychological therapies service reason’.^15^ However, the discharge codes used in the study databases were from version 1.5 of the IAPT data-set (July 2014) and were slightly different from the current codes that have been used since version 2.0 (October 2019). These differences are described in the version 2.0 NHS change specification for the data.^16^ Version 1.5 includes code ‘Completed scheduled treatment’, which was labelled 1, with all other codes labelled 0, including ‘Suitable for IAPT service, but the patient declined treatment that was offered’, ‘Dropped out of treatment (unscheduled discontinuation)’ and ‘Not suitable for IAPT service – signposted elsewhere by mutual agreement’. ‘Unknown’ and ‘NA’ comprised 4% of the data in total and were excluded as 4291 of 4589 such records were for 2022–2023, possibly because referrals for the latter year were still open when data were downloaded.

**Table 1:** Numbers and percentages of referrals by outcome categories (completed treatment or not, using codes from version 1.5 of the IAPT data-set,^16^ and recovered or not)

Treatment/recovery status	Number of referrals	Percentage of referrals (%)
Completed treatment or not		
Completed scheduled treatment	30 167	25.8
Suitable for IAPT service, but patient declined treatment that was offered	35 773	30.6
Dropped out of treatment (unscheduled discontinuation)	25 476	21.8
Not suitable for IAPT service – signposted elsewhere by mutual agreement	13 477	11.5
Discharged by mutual agreement following advice and support	6432	5.5
Referred to another therapy service by mutual agreement	2915	2.5
Not suitable for IAPT service – no action taken or directed back to referrer	1303	1.1
Referred to non-IAPT service	370	0.3
Other^b^	949	0.8
Total	116 945^a^	
Recovered or not^c^		
Recovered	17 437	54.5
Not recovered	14 584	45.5
Total assessed for recovery	32 021	26.3
Total not assessed for recovery	89 513	73.7
Total	121 534	

IAPT, Improving Access to Psychological Therapies.

a. Total does not include ‘not known‘ and ‘NA‘, *n* = 4589, of which 4291 were in 2022–2023.

b. Includes categories with less than 0.1%, such as ‘Deceased’ and spurious codes not included in the IAPT data-set (version 1.5).

c. All patients receiving two or more treatment sessions were assessed for recovery, not only patients completing scheduled treatment.

The ‘recovery’ indicator forms part of the national standards for IAPT and is defined in the NHS Talking Therapies for anxiety and depression manual.^2^ A patient was considered to have ‘recovered when they moved from above to below a pre-determined clinical threshold using paired scores from the nine-item Patient Health Questionnaire, a self-administered, depression symptom measure, and one of several other validated, condition-relevant questionnaires itemised in the Talking Therapies manual.^2,17^ Recovery could only be recorded from the second treatment session onwards and was therefore not available for patients receiving no treatment or one treatment session; however, it may have been recorded for patients that did not complete scheduled treatment but did have two treatment sessions, such as some patients that dropped out.

For the first part of the study, which investigated characteristics associated with recovery and treatment completion, only IAPT data were used. For the second part of the study, which describes further referrals, the IAPT referrals database was linked with the secondary mental health referrals database for the same period and geographic area using the unique patient reference (pseudonymised NHS number). To do this, simplified versions of each database containing only essential variables were created (patient ID, referral ID, referral date, external referral marker and service name), and variable names were harmonised. The combined database was pruned to retain only records for patients with an IAPT referral in 2019–2020 and only external referrals (rather than referrals between internal departments in a service). Referrals for each patient were ordered by referral date and indexed (numbered).

### Statistical analysis

R statistical software version 4.1.2 for Windows (R Core Team, R Foundation for Statistical Computing, Vienna, Austria; see https://www.r-project.org/) was used for all data management and statistical analyses. Odds ratios were used to quantify the strength of association between patient characteristics (age, gender, ethnicity and relative deprivation) and two dichotomous outcome variables, ‘recovered’ (yes/no) and ‘completed treatment’ (yes/no). We hypothesised that age, gender, ethnicity and deprivation could all influence patients’ recovery or completion of treatment, either directly or through unmeasured variables such as mental health states or healthcare utilisation and delivery. Previous research has found that lower rates of recovery in some ethnic groups were attenuated when deprivation was controlled for.^8^ Other research has shown that gender, age and deprivation may interact in predicting recovery rates.^6^ More generally, age and gender, and deprivation and ethnicity are frequently associated with each other and with health outcomes, and so crude estimates of their associations with health outcomes may be confounded. For this reason, in addition to reporting unadjusted odd ratios, we report mutually adjusted odds ratios (aORs) using multiple logistic regression models whereby age, gender, ethnicity and relative deprivation were included as covariates. The financial year was also included as a covariate to control for differences between financial years, such as potential changes to services because of the COVID-19 pandemic. Both odds ratios and aORs are reported, together with 95% confidence intervals, with a confidence interval crossing 1 considered to indicate a non-significant result.

Age categories included youth (16–17 years old) and young people (18–24 years old) to correspond to the structure of mental health services in N&W. Ethnicity was coded as ‘White British’, ‘Other’ (all other ethnicities) and ‘Not known’. In the IAPT database, trans-female (assigned male at birth) was recorded as female and trans-male (assigned female at birth) as male. Gender-fluid, non-binary and gender-neutral were recorded separately and form the ‘Other’ gender category. Fourteen records with missing gender were not included in the regression analysis. Deprivation was reported using decile rank, where decile 1 is the most deprived and 10 the least deprived.

**Table 2 tbl2:** Participants’ characteristics by outcome

Characteristic	Completed treatment or not		Recovered or not^a^	
	Number in category	Percentage completed	Number in category	Percentage recovered
Age category, years				
16–17	3146	21.0%	735	42.6%
18–24	23 563	18.5%	6074	46.9%
25–35	35 089	24.3%	9880	53.6%
36–70	50 510	30.5%	14 216	57.6%
>70	4637	26.3%	1117	70.7%
*P*-value^b^		<0.001		<0.001
Gender category				
Female	77 669	27.2%	22 221	53.9%
Male	38 712	23.2%	9633	56.1%
Other	564	17.2%	167	36.5%
*P*-value^b^		<0.001		<0.001
Ethnicity category				
White British	90 380	24.9%	25 866	54.7%
Other	8027	23.2%	2373	53.9%
Unknown	18 538	31.4%	3783	53.2%
*P*-value^b^		<0.001		0.183
Deprivation categor*y* ^c^				
IMD1	13 775	17.8%	3084	45.0%
IMD2	11 918	21.0%	3054	50.7%
IMD3	10 463	22.8%	2715	52.4%
IMD4	16 547	25.7%	4524	53.9%
IMD5	15 669	26.6%	4422	56.0%
IMD6	15 319	28.2%	4366	56.6%
IMD7	9609	29.1%	2754	57.1%
IMD8	9937	30.1%	2915	58.1%
IMD9	8182	31.0%	2510	57.8%
IMD10	5526	32.2%	1678	58.2%
*P*-value^b^		<0.001		<0.001
2019–2020	30 670	22.6%	3593	55.9%
2020–2021	24 283	30.0%	8852	56.0%
2021–2022	32 619	26.2%	11 444	53.1%
2022–2023	29 373	25.2%	8132	54.2%
*P*-value^b^		<0.001		<0.001
Total records	116 945	30167	86 778	32 021

a. Recovery status was assessed for patients that completed two or more treatment sessions and may have included patients that did not complete scheduled treatment, such as patients that dropped out.

b. Chi-squared test.

c. IMD1 (Index of Multiple Deprivation decile 1) is the most deprived decile.

**Table 3:** Factors associated with treatment completion: unadjusted odds ratios and mutually adjusted odds ratios (aORs) with 95% confidence intervals

Characteristic	Completed treatment or not unadjusted		Completed treatment or not adjusted	
	Odds ratio	95% CI	Odds ratio	95% CI
Age category, years				
36–70 (reference)	1.0		1.0	
16–17	0.61	0.56–0.66^a^	0.53	0.48−0.59^a^
18–24	0.52	0.50–0.54^a^	0.48	0.46−0.50^a^
25–35	0.73	0.71–0.76^a^	0.72	0.69−0.75^a^
>70	0.81	0.76–0.87^a^	0.72	0.66−0.78^a^
Gender category				
Female (reference)	1.0		1.0	
Male	0.81	0.78–0.83^a^	0.77	0.75−0.80^a^
Other	0.56	0.45–0.69^a^	0.72	0.55−0.93^a^
Ethnicity category				
White British (reference)	1.0		1.0	
Other	0.91	0.86–0.96^a^	0.94	0.88−1.01
Unknown	1.38	1.34–1.43^a^	1.46	1.40−1.52^a^
Deprivation decile				
1 (reference)	1.0		1.0	
2	1.23	1.16–1.30^a^	1.29	1.20−1.39^a^
3	1.37	1.29−1.45^a^	1.40	1.29−1.51^a^
4	1.60	1.51−1.69^a^	1.68	1.57−1.80^a^
5	1.68	1.59−1.77^a^	1.74	1.63−1.87^a^
6	1.81	1.72−1.92^a^	1.93	1.80−2.07^a^
7	1.90	1.78−2.04^a^	2.03	1.89−2.20^a^
8	1.99	1.87−2.08^a^	2.18	2.02−2.35^a^
9	2.08	1.95−2.20^a^	2.30	2.13−2.49^a^
10	2.19	2.04−2.33^a^	2.46	2.25−2.69^a^
Year				
2022–2023 (reference)	1.0		1.0	
2019–2020	0.86	0.83−0.90^a^	0.89	0.85–0.93^a^
2020–2021	1.27	1.22−1.32^a^	1.45	1.38–1.52^a^
2021–2022	1.05	1.02−1.09^a^	1.12	1.08–1.17^a^

a. Statistically significant confidence intervals.

As outcome variables were dichotomous, binary logistic regression analyses were used to identify the factors associated with being in one outcome response category or the other, e.g. ‘recovered’ or ‘not recovered’. As some individuals had more than one referral, mixed effects logistic regressions were employed, with the individual patient as the random effect (that is, with random variation in intercepts among individuals). All covariates were categorical. Reference categories in the logistic regression models were age group 36–70 years, female, White British ethnicity, decile 1 (most deprived) and financial year 2022–2023. Logistic regression was carried out using the lme4 package, selecting the ‘boyyqa’ optimiser.^18^ Chi-squared tests were used to assess the unadjusted associations between each regression covariate and dichotomous outcome variable[Table tbl2].

### Patient referral analysis

Patient referral pathways were analysed for patients who had a record for ‘recovered’ or ‘not recovered’ in 2019–2020, and patients with a record for ‘completed treatment’ or ‘not completed’ in 2019–2020. We tracked these patients to describe subsequent referrals received by either IAPT or secondary mental health services up to 31 March 2023.

We tabulated and described the proportions of recovered versus not recovered patients who had at least one further referral, as well as the proportions that had completed treatment versus not completed treatment who had at least one further referral (using chi-squared tests for independence to determine whether there was a statistically significant difference between the groups). We also took first further referrals for patients and tabulated the proportion of these received by IAPT versus the proportion received by secondary mental healthcare (again using chi-squared tests for independence).

R package networkD3 was used to construct Sankey diagrams illustrating patient re-referral pathways.^19^ Sankey diagrams are flow diagrams that represent quantitative information using ‘nodes’ (or boxes) whose sizes are based on the number of patients within them.^20^ We limited our description of re-referrals to a maximum of five to keep the Sankey diagrams legible and because only a very small percentage of patients had more than five further referrals.

### Ethical approval

Ethical approval was provided by the UEA Faculty of Medicine and Health Sciences Research Ethics Subcommittee [ETH2223-17290, 20 March 2023].

### Consent

This study used secondary data. A privacy notice was issued that data held by N&W ICB was being sublicensed to Norfolk County Council (NCC) for service evaluation and planning purposes.

## Results

### Treatment completion and recovery following an IAPT referral

Of 116 945 records for completion status, 25.8% (30 167) indicated that the patient had completed scheduled treatment (Table 1). Of those patients with records that indicated ‘not completed’, the largest groups were the 30.6% (35 773) of patients who were stated to be ‘Suitable for IAPT service, but patient declined treatment that was offered’ and the 21.8% (25 476) who ‘Dropped out of treatment (unscheduled discontinuation)’. A further 11.5% (13 477) were ‘Not suitable for IAPT service – signposted elsewhere by mutual agreement’. Over the study period, 26.3% (32 021) of referrals were assessed for recovery, of whom 54.5% (17 437) recovered.

Table 2 shows the percentages of cases by covariate group and outcome. Differences were observed within covariate groups by outcome, particularly by age group. For example, 18.5% of those aged 18–24 completed treatment, as opposed to 30.5% of those aged 36–70. Differences for recovery were even larger, with 42.6% of those aged 16–17 years and 70.7% of those aged >70 years having recovered. For deprivation decile, there was a steady upward trend from the most deprived decile (decile 1) to the least deprived for both treatment completion rates and recovery rates. Differences were less evident for ethnicity and for gender, apart from ‘other gender’ (gender-fluid, non-binary and gender-neutral).

Odds ratios, aORs and 95% confidence intervals are shown in Table 3 for treatment completion and in Table 4 for recovery. An odds ratio greater than 1 indicated that patients in a given category were more likely to recover or to complete treatment than those in the reference category, and an odds ratio of less than 1 indicated that they were less likely to recover or complete treatment.

**Table 4:** Factors associated with recovery: unadjusted odds ratios and mutually adjusted odds ratios (aORs) with 95% confidence intervals

Characteristic	Completed treatment or not unadjusted		Completed treatment or not adjusted	
	Odds ratio	95% CI	aOR	95% CI
Age category, years				
36–70 (reference)	1.0		1.0	
16–17	0.55	0.47–0.63^a^	0.53	0.46–0.63^a^
18–24	0.65	0.61–0.69^a^	0.64	0.60–0.69^a^
25–35	0.85	0.81–0.90^a^	0.85	0.80–0.90^a^
>70	1.78	1.56–2.03^a^	1.79	1.56–2.06^a^
Gender category				
Female (reference)	1.0		1.0	
Male	1.1	1.04–1.15^a^	1.09	1.04–1.15^a^
Other	0.49	0.36–0.68^a^	0.60	0.43–0.83^a^
Ethnicity category				
White British (reference)	1.0		1.0	
Other	0.97	0.89–1.06	1.06	0.97–1.16
Unknown	0.94	0.88–1.01	0.94	0.88–1.01
Deprivation decile				
1 (reference)	1.0		1.0	
2	1.26	1.14–1.38^a^	1.27	1.15–1.42^a^
3	1.34	1.21–1.48^a^	1.33	1.19–1.48^a^
4	1.43	1.30–1.57^a^	1.42	1.29–1.57^a^
5	1.55	1.41–1.71^a^	1.53	1.38–1.68^a^
6	1.59	1.45−1.76^a^	1.56	1.42−1.73^a^
7	1.62	1.46−1.84^a^	1.60	1.43−1.78^a^
8	1.69	1.53−1.83^a^	1.69	1.52−1.89^a^
9	1.67	1.50−1.83^a^	1.67	1.49−1.87^a^
10	1.70	1.51−1.89^a^	1.74	1.53−1.98^a^
Year				
2022–2023 (reference)	1.0		1.0	
2019–2020	1.07	0.99−1.16	1.11	1.02−1.20^a^
2020–2021	1.07	1.01−1.14^a^	1.12	1.05−1.20^a^
2021–2022	0.96	0.90−1.01	0.98	0.92−1.04

a. Statistically significant confidence intervals.

As shown in Table 3, referrals for patients of all other ages were less likely to complete treatment compared with those in the reference age group (36−70 years). Completion was especially low for those aged 16–17 years (aOR = 0.5, 95% CI: 0.5–0.6) and those aged 18–24 years (aOR = 0.5, 95% CI: 0.5–0.5). Males had lower odds of completing treatment than females (aOR = 0.8, 95% CI: 0.8–0.8), as did those of ‘Other’ gender (aOR = 0.7, 95% CI: 0.6–0.9). Those who were defined as ‘Unknown’ ethnicity had much higher odds of completing treatment compared with those defined as White British (aOR = 1.5, 95% CI: 1.4–1.5). Referrals for patients in deprivation decile 1 (the most deprived) had the lowest odds of completing treatment, and these odds increased steadily with decreasing deprivation.

Patients had higher odds of recovery in financial years 2020–2021 and 2021–2022 compared with 2022–2023, whereas odds of recovery were lower in 2019–2020.

As shown in Table 4, odds of being recovered increased with age, with aOR values of 0.5 (95% CI: 0.5–0.6) for referred patients aged 16–17 years and 1.8 (95% CI: 1.6–2.1) for those aged >70 years compared with the reference group (aged 36–70 years). Males were slightly more likely to attain recovered status compared with females (aOR = 1.1, 95% CI: 1.0–1.2), whereas those recorded as ‘Other’ gender had much lower odds (aOR = 0.6, 95% CI: 0.4–0.8). There were no differences for ethnicity. Referred patients in the most deprived areas had the lowest odds of being recovered, with odds increasing relatively steadily with reducing deprivation. Patients had higher odds of completion in financial years 2019–2020 and 2020–2021 than in 2022–2023.

Using adjusted odds in the multiple regression model resulted in minimal differences in odds ratios compared with the unadjusted odds.

### Further referral following an IAPT referral

The linked IAPT and secondary mental health referrals database consisted of 226 169 records for 134 178 patients. IAPT patients with referrals with the following four outcome states in 2019–2020 were identified: ‘completed treatment’ (*n* = 6513), or ‘not completed’ (*n* = 12 146), ‘recovered’ (*n* = 2007) and ‘not recovered’ (*n* = 1586). Summary data tables were created to identify numbers of subsequent referrals for these patients up to 31 March 2023 and whether the referral was to IAPT or secondary mental healthcare.

Patients that had not completed treatment in 2019–2020 had a higher percentage of further referrals to 31 March 2023 than those who had completed treatment (55.3% *v*. 45.4%; *P* < 0.001; Table 5). Patients who had not recovered had a higher percentage of further referrals than those who had recovered (53.1% *v*. 39.4%; *P* < 0.001; Table 5).

**Table 5:** IAPT referrals for 2019–2020 with at least one further referral up to 31 March 2023 and percentages of first further referrals to IAPT and secondary mental healthcare

Further referral status/desitination	Completed treatment or not^a^		Recovered or not	
	Completed (*n* = 6513)	Not completed (*n* = 9469)	Recovered (*n* = 2007)	Not recovered (*n* = 1586)
Percentage with a further referral				
Further referral	45.4%	55.3%	39.4%	53.1%
No further referral	54.6%	44.7%	60.6%	46.9%
*P*-value		<0.001		<0.001
For those with a further referral, destination of the first further referral				
IAPT	77.2%	56.4%	69.7%	60.6%
Secondary mental health	22.8%	43.6%	30.3%	39.4%
*P*-value		<0.001		<0.001

IAPT, Improving Access to Psychological Therapies.

a. Chi-squared test.

The primary route for all further referrals was to IAPT; however, for the first further referral, there was a relatively higher proportion of referrals to secondary mental health for those that had not completed compared with those that had completed treatment (43.6% *v*. 22.8%; *P* < 0.001; Table 5), and for those who had not recovered compared with those who had recovered (39.4% *v*. 30.3%; *P* < 0.001; Table 5).

**Fig. 1:**
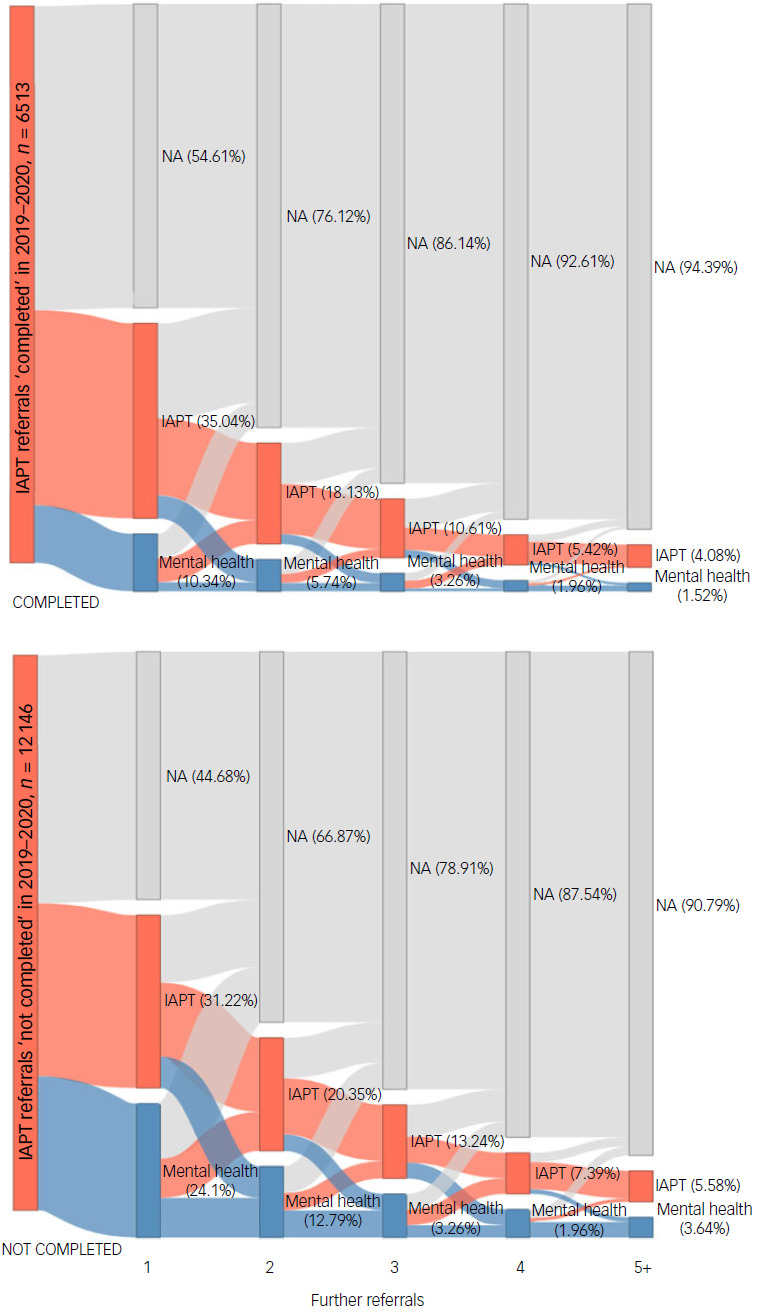
Sankey charts showing further referrals (up to five) for those referred to Improving Access to Psychological Therapies (IAPT) in 2019–2020 by ‘completed’ and ‘not completed’ status. Red boxes indicate referrals to IAPT, and blue boxes indicate referrals to secondary mental healthcare. Boxes marked ‘NA’ indicate the total percentage with no further referrals at that point.

The Sankey diagrams in Fig. 1 provide an illustration of the findings presented in Table 5 using cases for completed treatment and not completed. The first (left-hand) column in each represents patients in 2019–2020 with a completion status. The subsequent columns represent further referrals to 31 March 2023, from one further referral to five or more further referrals. For example, taking the column marked 1 for the ‘Completed treatment’ Sankey diagram, the box marked ‘NA’ shows that 54.6% (*n* = 3557) had no further referral up to 31 March 2023, 35% (*n* = 2230) had a further referral to IAPT and 10.3% (*n* = 673) had a further referral to secondary mental healthcare. Taking only those with a further referral in the column labelled 1 (*n* = 2954), rather than all cases, 77.2% had a referral to IAPT and 22.8% a referral to secondary mental healthcare; these latter figures are given in Table 4 to aid comparison between those who had completed and not completed treatment, and between those who had and had not recovered.

The Sankey diagrams also show that some patients had more than one further referral in the period of interest; for example, for those that completed treatment in 2019–2020, 23.9% of patients had two further referrals (to either IAPT or secondary mental healthcare), 13.9% had three further referrals, 7.4% had four further referrals and 5.6% had five or more further referrals.

## Discussion

### Treatment completion and recovery

In this study, more than half of eligible cases were recorded as ‘recovered’, but this represented a small proportion of total referrals, for example, 18.5% in 2021–2022. Young people were less likely to recover than those in older age groups. This was in line with nationally published data in which recovery rates for individuals aged 16–17 years were lower than for those aged 18+ years.^1^ Young people were also less likely to complete treatment. O’Keeffe et al (2019) investigated therapy dropout among children aged 11–17 years and identified three types of patient that dropped out of therapy: ‘got-what-they-needed‘ individuals, who stopped because they felt better; ‘dissatisfied’ individuals, who found therapy unhelpful or uncomfortable; and ‘troubled‘ individuals, who faced life instability.^21^ Those in the ‘got-what-they-needed‘ category showed better outcomes compared with those classified as ‘dissatisfied‘, as well as those who completed therapy, leading the authors to suggest that young people may benefit from a brief intervention and be able to self-determine their therapy end-point. The authors suggest that this typology of individuals who drop out provides a framework for managing different types of disengagement from treatment to reduce dropout rates among patients.

Around two-thirds of referrals to IAPT (66.5%) were for females, who were more likely to complete treatment. Males had slightly higher odds of recovery than females, in line with previous research.^7^ Individuals of ‘other’ gender (gender-fluid, non-binary and gender-neutral) had much lower odds of recovery than females. Watkinson et al (2024) recently described disproportionately high rates of self-reported mental health conditions among gender minorities, including some groups identifying as non-binary, indicating a need for better training of healthcare professionals to meet the needs of gender-diverse patients.^22^

Those living in the least deprived neighbourhoods had higher odds of both recovery and completion compared with those living in the most deprived, who had the lowest odds of recovery and completion. Poorer psychological therapy outcomes among socioeconomically deprived people have been identified in the research literature and nationally for IAPT services.^23,24^ Patients living in low-income areas may require higher numbers of treatment sessions to achieve recovery.^23^

Although the research literature suggests that patient characteristics such as age, ethnicity, deprivation and gender may be associated with each other and with health outcomes, mutually controlling for these characteristics resulted in only minor differences in odds ratios, indicating minimal confounding by covariates. However, it is possible that other variables not available in this study confounded associations identified between patient characteristics and outcomes.

### Further referral

In 2019–2020, 55.9% of patients were recorded as ‘recovered’; of these, 39.4% had subsequent referrals over the next 3–4 years, compared with 53.1% among those ‘not recovered’. Thus, even when therapies recommended by the National Institute of Health and Care Excellence were used, recovery may have been short term for many of the relatively small group of patients who both engaged with treatment and were recorded as having recovered. Assessment of whether a further referral rate of 39.4% was acceptable for those recorded as recovered was outside the scope of this study. Ali et al (2017) found that 53.1% of those who recovered following IAPT low-intensity cognitive–behavioural therapy had relapsed within the year.^10^ The higher figure reported by Ali et al (2017) may have been because their study considered a subset of IAPT patients receiving a specific treatment, but also because monthly depression and anxiety scores were used to identify relapse rather than further referral. An individual may decide not to seek help, or issues may resolve before help is sought in the form of a further referral; patients may also be referred to services other than IAPT or secondary mental healthcare.

Those who completed treatment had fewer further referrals than those that did not (45.4% *v*. 55.3%). The majority of those not completing treatment either declined services or dropped out, and regression results showed that non-completion was more common for certain groups. This presents a policy challenge, because referrals that result in the patient not engaging in treatment, particularly where there is subsequent further referral, affects not only IAPT resources but also those of referring organisations such as general practitioners. This is a missed opportunity to provide appropriate and timely intervention for patients.

A further finding was that for those recorded as ‘not completed’, relatively more further referrals were to secondary mental healthcare rather than IAPT compared with those recorded as ‘completed treatment’. This is an example of transition to secondary care that was identified as an area requiring further research by Martin et al in 2022. Some of these further referrals may have involved patients who were identified as ‘not suitable for IAPT service’ (Table 1); this may have been because IAPT is largely accessed by self-referral and may thus be easier to access than secondary healthcare services that require a referral from a professional. These findings suggest that there was a group of individuals who self-referred or were referred to IAPT, where either the delivery model or the treatment type was not suited to them. This may mean that services require adaptation for these groups or that there should be a route by which these referrals are made more quickly to secondary mental healthcare services.

### Strengths and limitations

A strength of this study was that the large sample size meant it was sufficiently powered for analysis of smaller subgroups, such as those of ‘Other’ gender. However, care should be taken when interpreting results for individuals of ‘Other’ gender, as these represent only 0.5% of referrals. There was a lack of categories for individuals whose gender was ‘trans’ in earlier years of the database, meaning that referrals in these groups could not be analysed separately. Ethnicity was categorised as ‘White British’ or ‘Other’. This approach was taken not only because there were several small, disparate groups, but also because of the high proportion of ‘Unknown’ (16%) ethnicity, which potentially affected the accuracy of the variable. We recognise that there may have been heterogeneity in associations between the subcategories within ‘Other’ and the outcome variables that this analysis could not detect. For 2022–2023, there were more ‘Unknown’ and ‘NA’ values for the variable ‘referral outcome’ (which was used to identify completion status) than in the preceding years (12% *v*. <1%), and approximately one-third fewer records for recovery status compared with the preceding 2 years, possibly because data were downloaded in mid-2023, meaning that referrals for the latter year were still open. There were also many fewer records for recovery status for 2019–2020 (32% of the following year), presumably owing to changes in protocols between these years. Recovery could not be measured for individuals who had undergone treatment but did not exceed clinical thresholds for anxiety and depression measures at the start of treatment; however, numbers of such individuals are low; for example, in 2022–2023, the figure for N&W was 4%.^1^ The data were collected by the mental health service providers, and there may have been problems with quality control or validity of outcome measures that the researchers were not aware of.

Although changes in completion and recovery rates over time were not the focus of this study, we controlled for financial year in the regression analysis; this was important given the potential effects of COVID-19 on service delivery. For example, the results indicated that during COVID-19 in 2020–2021, the odds of treatment completion were much higher (aOR 1.61), whereas the number of patients in the service was lower than in other financial years. We were not able to examine the independent effects of COVID-19 on recovery and completion (i.e. whether COVID-19 affected the demographics of those who recovered and/or completed treatment). COVID-19 may also have affected the results of the longitudinal analysis of further patient referrals.

Another strength of the study was that both IAPT and secondary mental health records over several years could be used for analysis of further referrals. However, we did not have data from other mental health providers, such as those in the voluntary sector; therefore, we could not describe the total number of further referrals. Neither could we describe whether referral in 2019–2020 was the first referral for a patient, whether a further referral was for the same condition, whether a patient left the area or died following a referral, or whether symptom severity was associated with further referral. Furthermore, the research was limited to one geographic area, and evidence suggests that there is variability in completion and recovery rates among service providers.^3,4^

### Policy implications

IAPT is an innovative service that has attracted international attention, with other countries implementing IAPT-like services as a result.^25,26^ Nationally, IAPT has been meeting ambitious access targets and in N&W, as in the rest of England, it is meeting its 50% recovery target. However, recovery is measured in only a small proportion of those referred, owing to high numbers of patients dropping out or declining service; many of these individuals will be re-referred to IAPT or secondary mental health services, with some having multiple further referrals. In addition, some groups such as children and young people, those living in deprived neighbourhoods and gender minorities have not only lower recovery rates but also lower completion rates, compounding disadvantage within the service. National standards from 2024 reflect a switch of focus from referral numbers to numbers completing treatment.^2^ It may also be advantageous to report recovery using an intention-to-treat approach, measuring the proportion that recover of all those starting treatment, rather than those completing two treatment sessions. This may encourage modifications in services for those most likely to drop out or decline services after their first session.

## Data Availability

Data are confidential pseudonymised medical records that are held by the Department of Public Health, NCC, and not by the authors, who are not authorised to legally distribute or share these data. Enquiries to access the data should be made to Public Health, Norfolk County Council, County Hall, Martineau Lane, Norwich, Norfolk, NR1 2DH (https://www.norfolk.gov.uk/).
